# Morphine analgesia in male inbred genetic diversity mice recapitulates the among‐individual variance in response to morphine in humans

**DOI:** 10.1002/ame2.12234

**Published:** 2022-06-03

**Authors:** Yin Yang, Bowen Guan, Qiang Wei, Wei Wang, Aimin Meng

**Affiliations:** ^1^ Key Laboratory of Human Disease Comparative Medicine, Ministry of Health, Beijing Engineering Research Center for Laboratory Animal Models of Human Critical Diseases, Institute of Laboratory Animal Sciences Chinese Academy of Medical Sciences (CAMS) and Peking Union Medical College (PUMC) Beijing China; ^2^ Department of Physiology and Neurobiology, School of Basic Medical Sciences Zhengzhou University Zhengzhou China

**Keywords:** inbred genetic diversity mice, morphine analgesia, morphine tolerance, quantitative trait loci mapping

## Abstract

Morphine is a widely used analgesic, but its use in clinical precision medicine is limited by the variance in response among individuals. Although previous studies have shown that individual differences in morphine can be explained in terms of pharmacodynamics and pharmacokinetics, genetic polymorphisms also play an important role. However, the genetic basis of different sensitivity and tolerance susceptibility to morphine remains ambiguous. Using 15 strains of inbred Genetic Diversity (GD) mice, a new resource with wide genetic and phenotypic variation, we demonstrated great variance in sensitivity to morphine analgesia and susceptibility to morphine tolerance between different GD strains. Among‐individual variance in response to morphine analgesia in the population can be modeled in GD mice. Two loci respectively may be associated with the among‐individual variance in morphine sensitivity and tolerance, confirming the role of genetic factors in among‐individual different responses to morphine. These results indicate that GD mice may be a potential tool for the identification of new biomarkers to improve the clinical administration of morphine.

## Graphical abstract

There is great variance in sensitivity to morphine analgesia and susceptibility to morphine tolerance among GD strains. Candidate genes Gm9966 and Tenm4 may be involved in this variance.

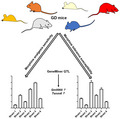

## 1 INTRODUCTION

Morphine, an opioid alkaloid, is an opioid receptor agonist that is effective in the management of most acute and chronic pain. Morphine directly inhibits the ascending afferent pathway of pain information originating from the dorsal horn of the spinal cord and activates the descending pain inhibition pathway originating from the midbrain to exert analgesic effect.^1^ However, chronic morphine administration usually leads to a significant decline in analgesic efficacy, also called tolerance. Furthermore, individual variation in sensitivity to morphine analgesia makes it hard to determine the dosage in pain management. These characteristics highlight the complexity and diversity in the mechanism of morphine analgesia and lead to many problems in clinical application.

Researchers reported that human genetic variability contributes to postoperative morphine consumption.^2^ Sensitivity to morphine is affected by genetic factors in terms of pharmacokinetics and pharmacodynamics. Variant of P‐glycoprotein encoding gene *ABCB1* regulates the transportation and distribution of morphine and affects its analgesic effect.^3^, ^4^ The diversity of UDP‐glucuronosyl transferase encoding gene *UGT2B7* also contributes to different responses to morphine pharmacokinetically.^5^ In contrast, variations of μ‐opioid receptor encoding gene *OPRM1* and catechol‐*O*‐methyltransferase encoding gene *COMT* regulate morphine‐induced downstream signaling and influence morphine analgesia pharmacodynamically.^6^, ^7^ In addition, great differences in susceptibility of morphine tolerance have also been found in different strains of mice or rats.^8^, ^9^ However, the genetic mechanism of morphine tolerance is complex and not yet completely understood. Tolerance may be associated with increased expression of P‐glycoprotein, making it difficult for morphine to cross the blood–brain barrier,^10^, ^11^ and increased orphanin and anti‐opioid peptide production, through their own receptors, antagonizing the effect of morphine.^12^, ^13^ In clinical research among different populations, investigation focused on genetic variation in response to morphine is abundant, but its in‐depth mechanism cannot be explored comprehensively owing to the ethical restrictions. However, clinical variation in sensitivity and susceptibility of tolerance to morphine is difficult to model in common laboratory animals owing to their limited genetic diversity, especially with inbred strains of mice or rats.

In this study, we investigated the genetic factors of individual variance to morphine analgesia and susceptibility to morphine tolerance by using 15 strains of mice from inbred Genetic Diversity (GD) cohort, which have almost the same genetic background as Collaborative Cross (CC) mice. This resource was developed from the breeding of CC mice, which were generated by crossing 8 diverse founder strains of mice (A/J, C57BL/6J, 129S1/SvImJ, NOD/LtJ, NZO/H1LtJ, CAST/Ei, PWK/PhJ, and WSB/EiJ). These mice together represent 90% of the common genetic variation in *Mus musculus* species.^14^ After more than 10 years of breeding, the resulting inbred CC strains exhibit a wide range of traits and have great advantages in integrative analysis of complex systems that can be defined only in vivo, such as resistance to Ebola virus infection and variability in immunoglobulin glycosylation.^15^, ^16^ Susceptibility to diseases, development of the animal model, and related genetic loci mapping are being investigated increasingly in CC mice.^17^, ^18^, ^19^ In our research, we found that different strains of GD mice exhibit an extremely wide range of variance in response to morphine analgesia, including large differences in morphine sensitivity and susceptibility of morphine tolerance.

## 2 MATERIALS AND METHODS

### 2.1 Animals

GD mice were provided by Institute of Laboratory Animal Sciences, Chinese Academy of Medical Sciences (CAMS) & Peking Union Medical College (PUMC). Mice were housed in individual ventilated cages in a barrier system on a 12‐h light/dark cycle with access to food and water ad libitum. All animal experiments were performed under the guidelines of the Animal Care and Use Committee of our Institute, and the IACUC number is MAM‐16001.

To exclude the effects of physiological cycle of female mice, adult male GD mice (8–12 weeks) were used. The mice were adapted to their living environment for 1 week and were handled at least 5 min per day continuously for 3 days before the start of experiments. The 15 strains of GD mice we used were BEM (CC032/GeniUnc) (*n* = 4), BOM (CC042/GeniUnc) (*n* = 4), BOON (CC008/GeniUnc) (*n* = 6), DAVIS (CC012/GeniUnc) (*n* = 4), FEW (CC025/GeniUnc) (*n* = 3), GIG (CC013/GeniUnc) (*n* = 4), LAM (*n* = 4), LAT (*n* = 2), LOT (*n* = 5), LOX (*n* = 3), NUK (CC010/GeniUnc) (*n* = 8), PEF (*n* = 3), PIPING (CC043/GeniUnc) (*n* = 4), SAT (CC016/GeniUnc) (*n* = 5), and TOP (CC023/GeniUnc) (*n* = 4).

### 2.2 Heat‐induced tail‐flick latency tests

Heat‐induced tail‐flick latency (TFL) tests were used to evaluate the thermal pain threshold of the mice. The tests were performed as previously described.^20^ In brief, restrainers were made to hold the mice while testing. The mice were handled and habituated to freely enter the restrainer. TFLs were tested before morphine injection. Using a super thermostatic circulating water bath (HH‐501, Jintan, Changzhou, China), the temperature of the water was maintained at 48°C. The mice were held in the restrainer. Two‐thirds of their tail was dipped in the water, and the latency of the tail withdrawal was measured. A cutoff time of 15 s was set to avoid tissue damage. The test was repeated 3 times with a 5‐min interval, and the pain threshold for each mouse was determined as the mean value of 3 TFL tests.

For morphine analgesic effect tests, TFLs were tested 30, 60, 90, and 120 min after each morphine injection. The pain threshold changes were presented as percentage of maximum probable effect (%MPE).

$$\begin{array}{c} \%\mathrm{MPE}=\left(\text{test}\;\mathrm{TFL}-\text{baseline}\;\mathrm{TFL}\right)/\\ \left(\text{cutoff time}-\text{baseline}\;\mathrm{TFL}\right)\times 100\%. \end{array}$$

The time‐%MPE curve was plotted, and morphing analgesic effects were presented as area under the curve (AUC). Susceptibility to morphine tolerance was estimated as percentage of decreased AUC of %MPE on the 6th day of morphine injection compared with the 1st day.

### 2.3 Drug administration

After baseline tests of the pain threshold, morphine hydrochloride (Northeast Pharmaceutical, Shenyang, China) was injected (2 mg/kg) subcutaneously before the analgesic effect tests. For morphine tolerance induction, morphine (2 mg/kg) was injected daily (once per day) for 6 days continuously. On the 1st day and 6th day of morphine injection, thermal pain thresholds at 30, 60, 90, and 120 min post‐morphine exposure were tested.

### 2.4 Quantitative trait locus mapping

Quantitative trait locus mapping was performed using GeneMiner (http://www.sysgen.org/Geniad2/), on which the genotype data are specific to the CC strains, as previously reported in the literature.^16^ MugaQTL method was used, and means of AUC of day 1 %MPE were used to analyze the strain sensitivity to morphine. Mean AUC percentage decrease of day 6 %MPE was compared with that of day 1 to analyze susceptibility to morphine tolerance. Logarithm of odds ratio (LOD) plots were generated, and a 2‐LOD drop interval from the peak position was calculated as the significant threshold of genetic locus of the relevant phenotype.

### 2.5 Statistics

Data were presented as mean ± standard error of the mean (SEM). Plot generation, column statistics, and AUC calculation were performed with GraphPad Prism 8.0 for Windows (GraphPad Software, Inc., USA). One‐way analysis of variance (ANOVA) followed by multiple comparisons with Bonferroni correction and *t*‐test was used to analyze the significance of differences. *p* < .05 was considered significant.

## 3 RESULTS

### 3.1 Variability of heat‐induced pain threshold in GD mice

**FIGURE 1:**
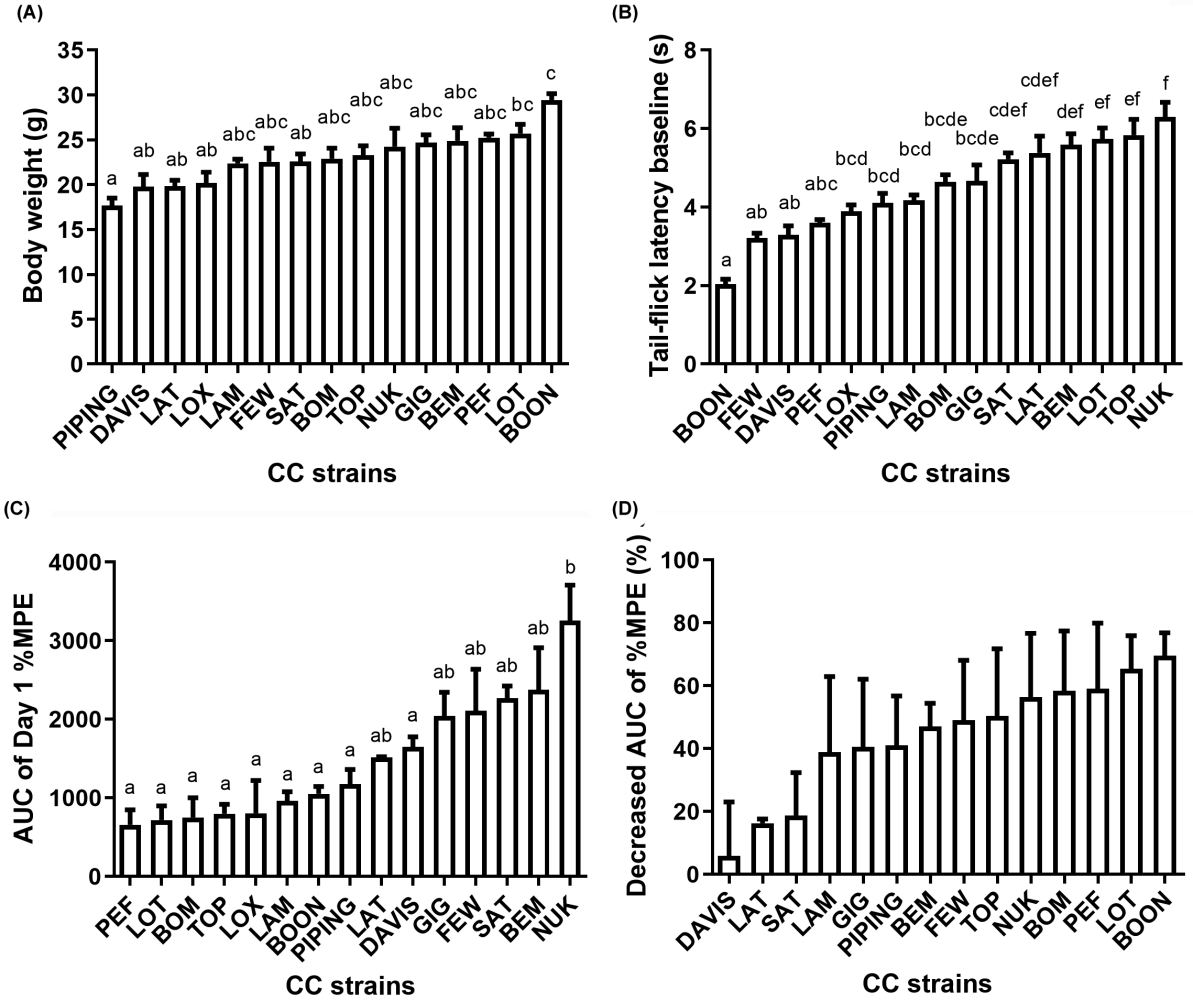
Variability in morphine sensitivity and tolerance susceptibility of the 15 strains of GD mice. (A) Body weight of each strain of GD mice. (B) Tail‐flick latency induced by 48°C water of each GD mice in naïve state. (C) Area under the time‐%MPE curve (AUC) of each strain of GD mice on day 1 of morphine injection were calculated and plotted, representing the sensitivity to morphine. (D) Percentage of decrease of AUC on day 6 compared with day 1 of morphine injection, representing the tolerance susceptibility to morphine analgesia. Data were represented as mean ± SEM, *n* = 2–8 (details of *n* values are shown in Materials and Methods). One‐way ANOVA followed by multiple comparisons with Bonferroni correction. Compact letter display was used to present the significant differences between groups from multiple comparisons. Those marked with different lowercase letters indicate significant differences between groups, and those marked with the same lowercase letters indicate no significant differences between groups. *p* values of the multiple comparisons were provided in the Supporting Information

Different strains of GD mice in the same week periods of age (8–12 weeks) exhibited a large variance of body weight, ranging from 17.65 ± 0.85 g (PIPING) to 29.40 ± 0.76 g (BOON) (Figure 1A), indicating great differences among GD strains in growth process. To determine whether basic pain threshold variation exists in different strains of GD mice, we tested the tail‐flick latencies of the 15 strains of GD mice in naïve state. In 48°C water tail immersion, tail‐flick latency of BOON was the shortest, 2.04 ± 0.12 s. In contrast, tail‐flick latency of NUK was 6.30 ± 0.37 s, more than 3‐fold longer than BOON (Figure 1B). These results demonstrate that GD mice have a wide range of traits, including sensitivity to pain stimulation.

### 3.2 Strain variation in response to morphine

**FIGURE 2:**
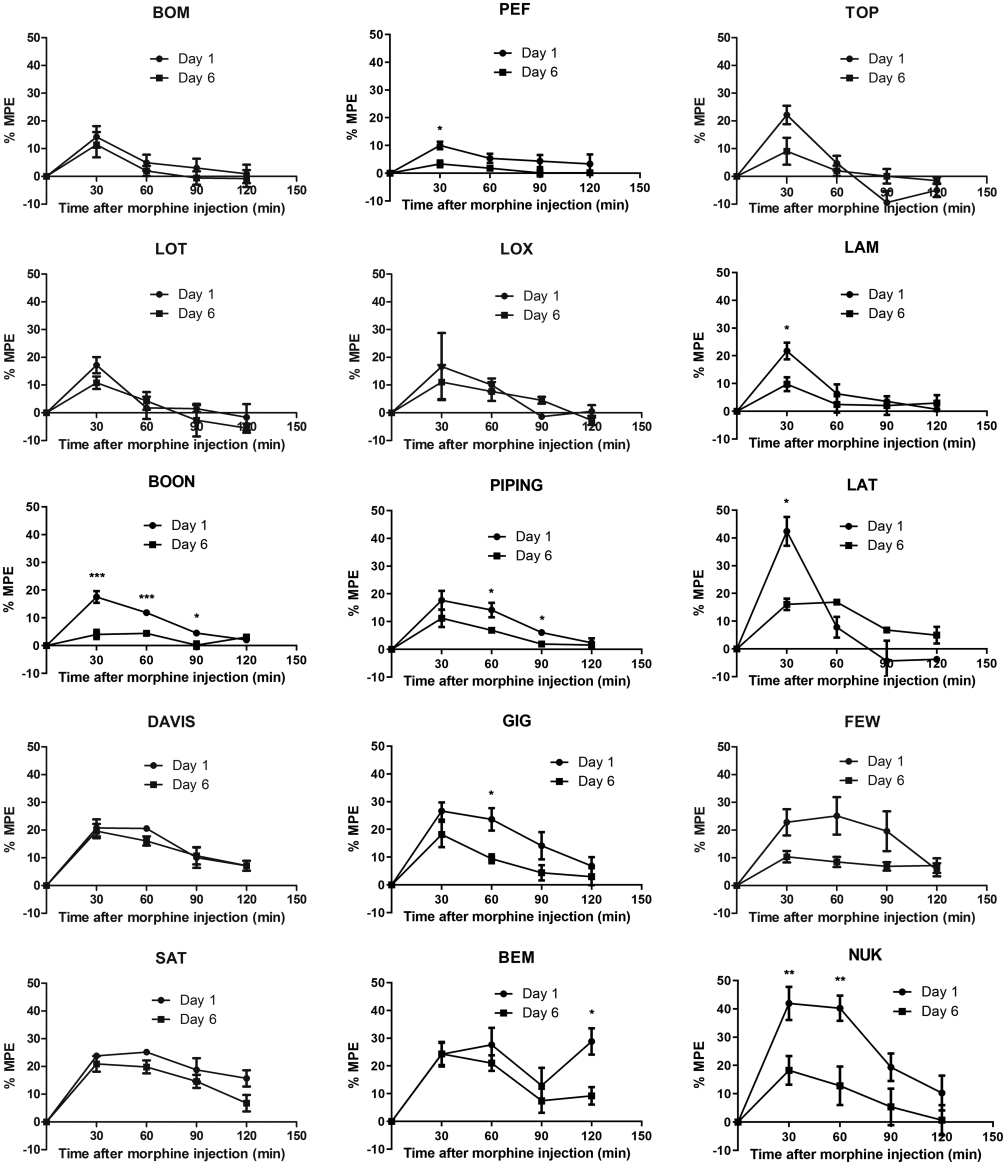
Morphine analgesic effect of each GD strains on days 1 and 6. Analgesic effect of morphine was evaluated by percentage of maximum probable effect (%MPE). Morphine (2 mg/kg) was continuously injected for 6 days. Analgesic effect of baseline, 30, 60, 90, and 120 min after morphine injection on days 1 and 6 were tested. Time‐%MPE curves were plotted. Data are represented as mean ± SEM, *n* = 2–8. *t*‐test of days 1 and 6 at each timepoint after morphine injection. *p < .05, **p < .01, ***p < .001

To characterize the responses of GD mice to morphine analgesia, the time‐%MPE curve of each strain of GD mice was plotted (Figure 2). %MPE represents the analgesic effect of morphine. On the first day of morphine injection, all GD mice exhibited the maximum analgesic effect 30 min after injection, except for FEW, SAT, and BEM, whose maximum %MPE was observed 60 min after injection. Analgesic effect of morphine gradually decreased over time and almost disappeared 120 min after morphine injection. Among the 15 strains of GD mice, transiently maximum analgesic effect was found in LAT (%MPE 42.36%) and NUK (%MPE 41.91%) 30 min after morphine injection on the first day. However, in LAT mice, analgesic effect decreased sharply, while in NUK mice, it was maintained for 90 min. In contrast, the minimum analgesic effect was observed in PEF (%MPE 10.03%) 30 min after the first morphine injection (Figure 2).

Compared with the first morphine injection, after continuous injection for 6 days, analgesic effect of morphine decreased in almost all the GD mice on the 6th day. However, in BOM, LOT, and DAVIS, the decrease was not obvious (Figure 2). These results prove that different strains of GD mice have different responses to morphine analgesia, and indicate that GD mice may be a promising resource to model the individual variance in the response to morphine use clinically.

### 3.3 Strain variation in sensitivity to morphine and susceptibility to morphine tolerance

To comprehensively evaluate the individual sensitivity to morphine and susceptibility to morphine tolerance, taking both the intensity and duration of analgesia effect into account, we calculated the area under the curve (AUC) of the time‐%MPE graph. The AUC of morphine injection on the first day represents the total analgesic effect of morphine. As Figure 1C shows (and Figure 2, for transient maximum analgesic effect), NUK is the strain most sensitive to morphine analgesia (AUC 3258 ± 448.7), while PEF is the strain least sensitive to morphine analgesia (AUC 658.5 ± 190.0). It is worth noting that AUC of LAT mice (AUC 1514 ± 10.50), whose transient maximum analgesic effect was also the strongest in the 15 strains, was only half that of the NUK mice (Figure 1C). This also reflects that use of morphine analgesia in LAT mice is not favorable.

To examine the susceptibility to morphine tolerance, we calculated the percentage decrease of AUC after morphine tolerance induction by continuous morphine injection for 6 days. As Figure 1D shows, total analgesic effect (AUC) decreased the most in BOON mice (69.50% ± 7.361%), demonstrating that BOON mice have the highest susceptibility to morphine tolerance. DAVIS (12.62% ± 17.12%) and LAT (16.31% ± 1.274%) mice exhibited the lowest decrease of AUC. However, there was only a trend of change in susceptibility to morphine tolerance among the strains but without significant differences. This is probably due to the limited number of strains used and the limited number of animals per strain. We had excluded the data on LOX mice since there were only 3 mice and the data had high standard error. These results indicate that DAVIS and LAT mice were less likely to develop morphine tolerance, while BOON mice were the most susceptive to morphine tolerance (Figure 1D).

### 3.4 Quantitative trait locus analysis

**FIGURE 3:**
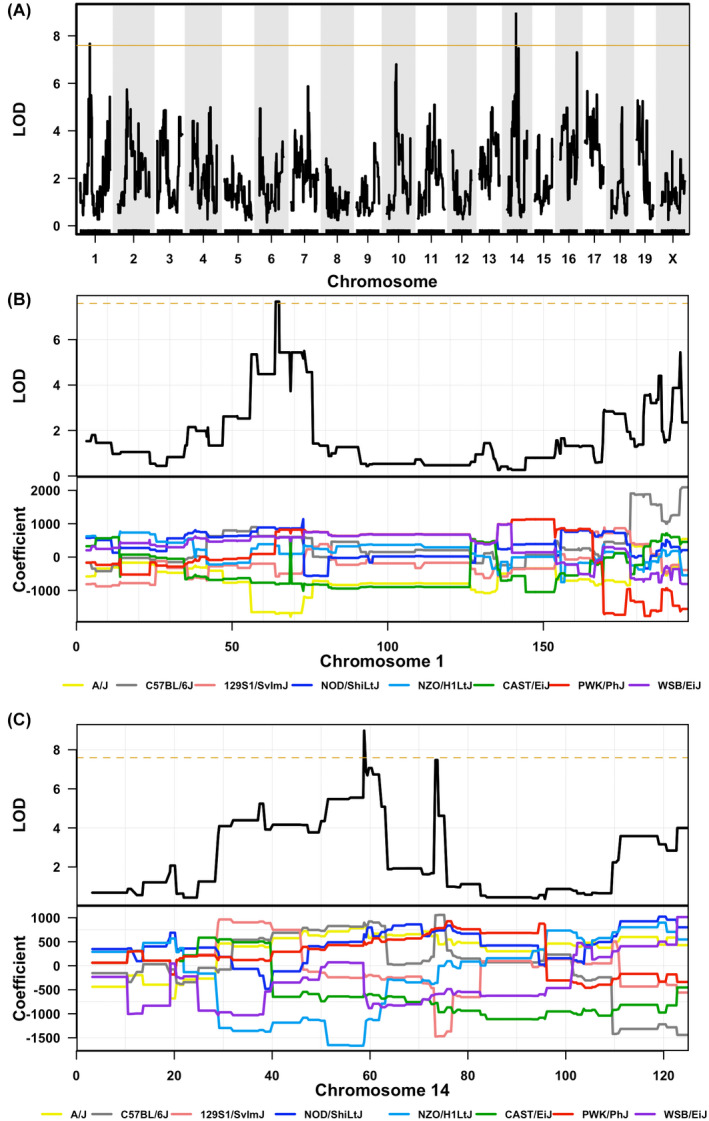
QTL mapping of morphine sensitivity. (A) QTL mapping of morphine sensitivity showing linkage to fragments in chromosome 1 and chromosome 14. (B) and (C) In details of each chromosome, accurate fragment positions and the standardized regression (β) coefficients of the 8 founder strains are demonstrated. The brown line is the permutation‐based genome‐wide 37% significance threshold. *n* = 15 biologically independent GD strains

Considering the great variance in the response to morphine analgesia in GD mice, to map the genetic loci of this phenotype variation, we performed QTL analysis using GeneMiner, a web application for QTL analysis using CC mice. For genetic factors underlying sensitivity to morphine, the results are shown in Figure 3. Fragment of 64.043 Mbp to 65.214 Mbp located on chromosome 1 (LOD 7.7) and fragment of 58.801 Mbp to 59.182 Mbp located on chromosome 14 (LOD 9.0) were associated with the variance in sensitivity to morphine analgesia (Figure 3A). Coefficient factors from chromosome 1 indicated that genetic information from C57BL/6J, NOD/ShiLtJ, NZO/H1LtJ, PWK/PhJ, and WSB/EiJ contribute to sensitivity to morphine, whereas genetic information from A/J mice made the individual not sensitive to morphine (Figure 3B). However, on chromosome 14, genetic information from A/J, C57BL/6J, NOD/ShiLtJ, and PWK/PhJ contributed to sensitivity to morphine, whereas genetic information from NZO/H1LtJ made the mice not sensitive to morphine (Figure 3C).

**FIGURE 4:**
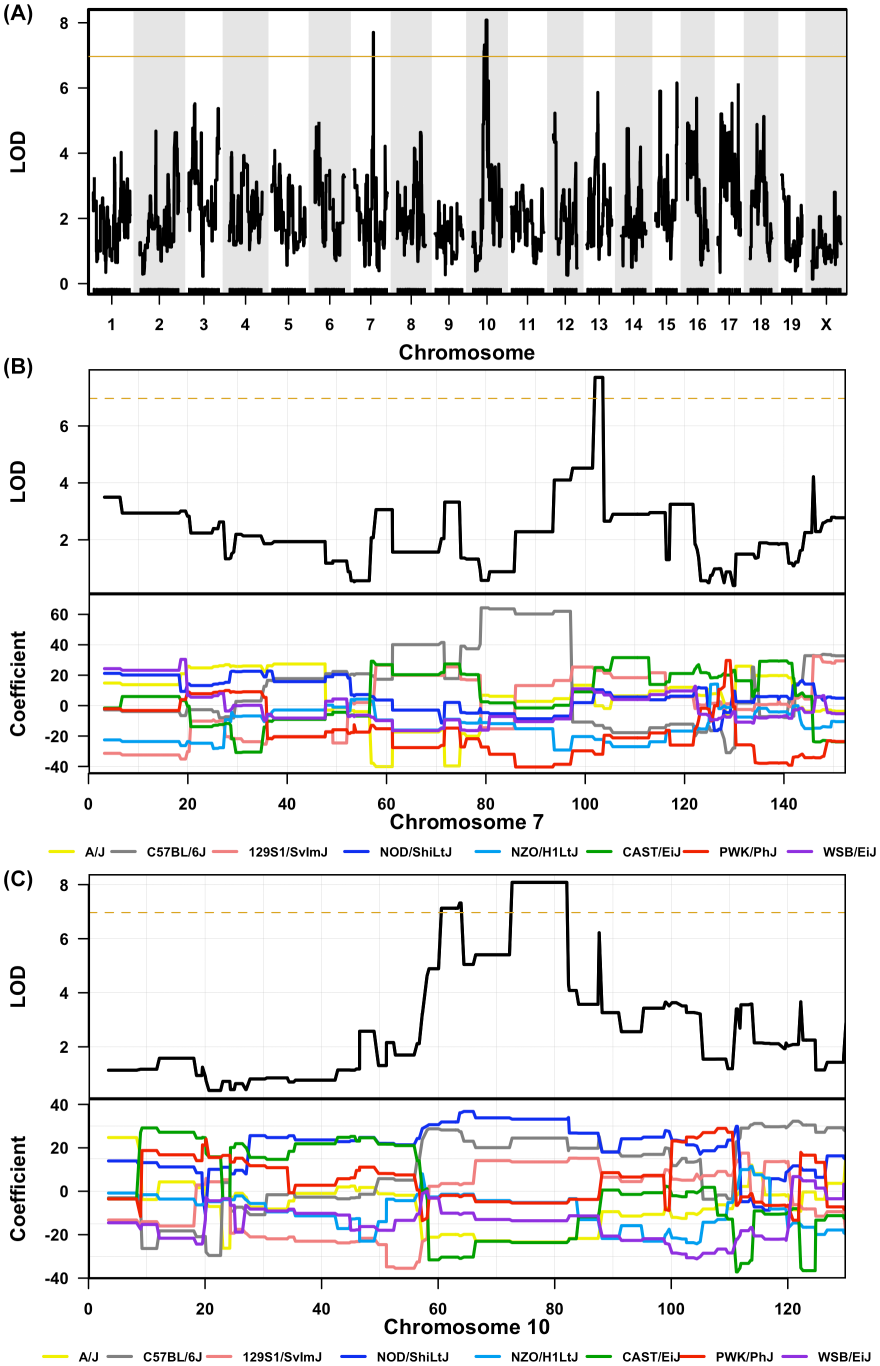
QTL mapping of susceptibility to morphine tolerance. (A) QTL mapping of susceptibility to morphine tolerance showing linkage to fragments in chromosome 7 and chromosome 10. (B) and (C) In details of each chromosome, accurate fragment positions and the standardized regression (β) coefficient of the 8 founder strains are demonstrated. The brown line is the permutation‐based genome‐wide 37% significance threshold. *n* = 15 biologically independent GD strains

Regarding susceptibility to morphine tolerance, 2 loci were found on chromosomes 7 and 10 (Figure 4A). Fragment from 101.983 Mbp to 103.817 Mbp located on chromosome 7 (LOD 7.7) was associated with morphine tolerance (Figure 4B). According to GeneMiner analysis, *Gm9966* gene and a single‐nucleotide polymorphism (SNP, rs31544069, a splice site) of gene *Tenm4* in this fragment from PWK/PhJ may make the mice less likely to develop morphine tolerance. Fragment from 60.567 Mbp to 82.371 Mbp located on chromosome 10 (2 peaks, LOD 7.1 and 8.1, respectively) was also associated with susceptibility to morphine tolerance (Figure 4C). In this fragment, genetic information from C57BL/6J, 129S1/SvImJ, and NOD/ShiLtJ made the mice more likely to develop morphine tolerance. However, genetic information from A/J, CAST/Ei, and WSB/EiJ made the mice less likely to develop morphine tolerance (Figure 4C).

## 4 DISCUSSION

Based on the existing investigations, individual responses to morphine analgesia are controlled by multiple genes related to pharmacokinetics and pharmacodynamics, such as *ABCB1*, *UGT2B7*, *OPRM1*, and *COMT*. In clinical research, individual variant responses to morphine analgesia are abundant and large variations have already been found.^21^, ^22^ Further investigation is lacking clinically because of ethical issues. High‐diversity CC mice populations are emerging to make up for the defect of genetic diversity in common inbred mice. For example, systemic virus infection revealed diverse CD8 T‐cell responses among different strains of CC mice.^23^ New mouse models of seizure susceptibility were identified with CC mice, and associated gene loci were mapped.^24^ Phenotypes of response to morphine are also diverse in inbred GD strains, according to our present study.

Previous work has reported that 9 SNPs in *ESR1*, *OPRM1*, and *COMT* can explain only 10.7% of variance of postoperative morphine consumption.^2^ Comparative studies using animal models with different genetic background are few. A study using 11 inbred mouse strains has reported that 129P3/J and LP strains display no evidence of tolerance development.^8^ However, in this study, we identified 129S1/SvImJ as morphine‐tolerance‐susceptive mice (Figure 4B,C). This may be due to the different characterization of genetics among 129 substrains. Recent research identified 129S1/SvImJ and 129P3/J as low‐ and high opioid‐taking substrains, respectively.^25^ In addition, C57BL/6J mice are more likely to develop morphine tolerance than DBA2/J mice. Dicer and H19 may be candidate genes involved in the variant response to chronic morphine administration.^26^ This is consistent with our findings since we also identified that genetic information from C57BL/6J mice made the GD mice more likely to develop morphine tolerance (Figure 4B,C). Research using more strains of inbred mice (23 strains) illustrated that multiple PDZ domain gene *Mpdz* may contribute to the inter‐strain variability in morphine tolerance.^27^ *Mpdz* gene is located on chromosome 4 in *Mus musculus*. However, in the present study, we did not identify any locus on chromosome 4 that was associated with morphine tolerance, which may be due to the limited number of available GD strains. Additional studies using different strains of rats suggests that Sprague–Dawley rats are more sensitive and less likely to develop tolerance to morphine than Wistar rats.^9^ In our study, we have found great differences in response to morphine analgesia in different strains of GD mice. In recent years, CC mice were used in many research areas, including pharmacology, toxicology, infection, and metabolic disorders,^28^, ^29^, ^30^, ^31^ highlighting their broad application prospects.

In this study, we have mapped the fragment of 60.567 Mbp to 82.371 Mbp located on chromosome 10 that was associated with development of morphine tolerance (Figure 4A). The main target of morphine, μ‐opioid receptor of *Mus musculus*, encoding gene *OPRM1* is located in this region (6 758 593–7 038 209 in chromosome 10 according to NCBI Gene database). This result confirms the credibility of using GD mice to map the loci associated with morphine tolerance. We also identified 2 candidate genes that may influence the susceptibility to morphine analgesia in mice, *Gm9966* and *Tenm4*. *Gm9966* is the predicted gene 9966. *Tenm4* gene encodes a protein named teneurin transmembrane protein 4 associating with proper neuronal connectivity during development. Increased variants in *Tenm4* may be associated with schizophrenia.^32^ In patients with essential tremor, variations in *Tenm4* were also shown by exome sequencing.^33^ The relationship of *Tenm4* mutations and morphine tolerance may be worth researching in future investigations. However, owing to the limited number of GD strains used in this study, the QTL analysis is not obviously significant.^34^, ^35^ To confirm the exact role of these genetic loci in individual differences in morphine analgesia, much work remains to be done and more strains of GD mice need to be tested following this preliminary work. Meanwhile, GD mice may play an increasingly important role as a new resource in the study of individual differences in drug responses.

## AUTHOR CONTRIBUTIONS

Yin Yang and Aimin Meng conceived the research. Yin Yang performed the experiments. Bowen Guan and Qiang Wei analyzed the data. Yin Yang drafted the manuscript. Wei Wang and Aimin Meng revised the manuscript. All authors have read and confirmed the manuscript.

## CONFLICT OF INTEREST

The authors declare that there is no conflict of interest.

## Supporting information


**Appendix S1** Supporting Information
