# Supplementary material for: Morphine analgesia in male inbred genetic diversity mice recapitulates the among‐individual variance in response to morphine in humans
Source: Animal Model Exp Med. 2022 Jun 3;5(3):288–96. doi: 10.1002/ame2.12234 (PMC9240740; doi:10.1002/ame2.12234)
Supplement: Supplementary file 1 — Appendix S1 Supporting Information [file AME2-5-288-s001.docx]

**Figure 1A**

| Number of families | 1 |  |  |  |  |
| --- | --- | --- | --- | --- | --- |
| Number of comparisons per family | 105 |  |  |  |  |
| Alpha | 0.05 |  |  |  |  |
|  |  |  |  |  |  |
| Bonferroni's multiple comparisons test | Mean Diff. | 95.00% CI of diff. | Significant? | Summary | Adjusted P Value |
| PIPING vs. DAVIS | -2.150 | -9.867 to 5.567 | No | ns | >0.9999 |
| PIPING vs. LAT | -2.200 | -11.65 to 7.251 | No | ns | >0.9999 |
| PIPING vs. LOX | -2.550 | -10.89 to 5.785 | No | ns | >0.9999 |
| PIPING vs. LAM | -4.750 | -12.47 to 2.967 | No | ns | >0.9999 |
| PIPING vs. FEW | -4.917 | -13.25 to 3.418 | No | ns | >0.9999 |
| PIPING vs. SAT | -4.950 | -12.27 to 2.371 | No | ns | >0.9999 |
| PIPING vs. BOM | -5.250 | -12.57 to 2.071 | No | ns | >0.9999 |
| PIPING vs. TOP | -5.650 | -13.37 to 2.067 | No | ns | 0.8942 |
| PIPING vs. NUK | -6.613 | -13.30 to 0.07048 | No | ns | 0.0565 |
| PIPING vs. GIG | -7.050 | -14.77 to 0.6668 | No | ns | 0.1332 |
| PIPING vs. BEM | -7.250 | -14.97 to 0.4668 | No | ns | 0.0997 |
| PIPING vs. PEF | -7.583 | -15.92 to 0.7518 | No | ns | 0.1389 |
| PIPING vs. LOT | -8.050 | -15.37 to -0.7292 | Yes | * | 0.0154 |
| PIPING vs. BOON | -11.75 | -18.79 to -4.706 | Yes | **** | <0.0001 |
| DAVIS vs. LAT | -0.05000 | -9.501 to 9.401 | No | ns | >0.9999 |
| DAVIS vs. LOX | -0.4000 | -8.735 to 7.935 | No | ns | >0.9999 |
| DAVIS vs. LAM | -2.600 | -10.32 to 5.117 | No | ns | >0.9999 |
| DAVIS vs. FEW | -2.767 | -11.10 to 5.568 | No | ns | >0.9999 |
| DAVIS vs. SAT | -2.800 | -10.12 to 4.521 | No | ns | >0.9999 |
| DAVIS vs. BOM | -3.100 | -10.42 to 4.221 | No | ns | >0.9999 |
| DAVIS vs. TOP | -3.500 | -11.22 to 4.217 | No | ns | >0.9999 |
| DAVIS vs. NUK | -4.463 | -11.15 to 2.220 | No | ns | >0.9999 |
| DAVIS vs. GIG | -4.900 | -12.62 to 2.817 | No | ns | >0.9999 |
| DAVIS vs. BEM | -5.100 | -12.82 to 2.617 | No | ns | >0.9999 |
| DAVIS vs. PEF | -5.433 | -13.77 to 2.902 | No | ns | >0.9999 |
| DAVIS vs. LOT | -5.900 | -13.22 to 1.421 | No | ns | 0.4237 |
| DAVIS vs. BOON | -9.600 | -16.64 to -2.556 | Yes | *** | 0.0006 |
| LAT vs. LOX | -0.3500 | -10.31 to 9.612 | No | ns | >0.9999 |
| LAT vs. LAM | -2.550 | -12.00 to 6.901 | No | ns | >0.9999 |
| LAT vs. FEW | -2.717 | -12.68 to 7.246 | No | ns | >0.9999 |
| LAT vs. SAT | -2.750 | -11.88 to 6.381 | No | ns | >0.9999 |
| LAT vs. BOM | -3.050 | -12.18 to 6.081 | No | ns | >0.9999 |
| LAT vs. TOP | -3.450 | -12.90 to 6.001 | No | ns | >0.9999 |
| LAT vs. NUK | -4.413 | -13.04 to 4.215 | No | ns | >0.9999 |
| LAT vs. GIG | -4.850 | -14.30 to 4.601 | No | ns | >0.9999 |
| LAT vs. BEM | -5.050 | -14.50 to 4.401 | No | ns | >0.9999 |
| LAT vs. PEF | -5.383 | -15.35 to 4.579 | No | ns | >0.9999 |
| LAT vs. LOT | -5.850 | -14.98 to 3.281 | No | ns | >0.9999 |
| LAT vs. BOON | -9.550 | -18.46 to -0.6394 | Yes | * | 0.0215 |
| LOX vs. LAM | -2.200 | -10.54 to 6.135 | No | ns | >0.9999 |
| LOX vs. FEW | -2.367 | -11.28 to 6.544 | No | ns | >0.9999 |
| LOX vs. SAT | -2.400 | -10.37 to 5.570 | No | ns | >0.9999 |
| LOX vs. BOM | -2.700 | -10.67 to 5.270 | No | ns | >0.9999 |
| LOX vs. TOP | -3.100 | -11.44 to 5.235 | No | ns | >0.9999 |
| LOX vs. NUK | -4.063 | -11.45 to 3.326 | No | ns | >0.9999 |
| LOX vs. GIG | -4.500 | -12.84 to 3.835 | No | ns | >0.9999 |
| LOX vs. BEM | -4.700 | -13.04 to 3.635 | No | ns | >0.9999 |
| LOX vs. PEF | -5.033 | -13.94 to 3.877 | No | ns | >0.9999 |
| LOX vs. LOT | -5.500 | -13.47 to 2.470 | No | ns | >0.9999 |
| LOX vs. BOON | -9.200 | -16.92 to -1.483 | Yes | ** | 0.0050 |
| LAM vs. FEW | -0.1667 | -8.502 to 8.168 | No | ns | >0.9999 |
| LAM vs. SAT | -0.2000 | -7.521 to 7.121 | No | ns | >0.9999 |
| LAM vs. BOM | -0.5000 | -7.821 to 6.821 | No | ns | >0.9999 |
| LAM vs. TOP | -0.9000 | -8.617 to 6.817 | No | ns | >0.9999 |
| LAM vs. NUK | -1.863 | -8.545 to 4.820 | No | ns | >0.9999 |
| LAM vs. GIG | -2.300 | -10.02 to 5.417 | No | ns | >0.9999 |
| LAM vs. BEM | -2.500 | -10.22 to 5.217 | No | ns | >0.9999 |
| LAM vs. PEF | -2.833 | -11.17 to 5.502 | No | ns | >0.9999 |
| LAM vs. LOT | -3.300 | -10.62 to 4.021 | No | ns | >0.9999 |
| LAM vs. BOON | -7.000 | -14.04 to 0.04448 | No | ns | 0.0538 |
| FEW vs. SAT | -0.03333 | -8.003 to 7.937 | No | ns | >0.9999 |
| FEW vs. BOM | -0.3333 | -8.303 to 7.637 | No | ns | >0.9999 |
| FEW vs. TOP | -0.7333 | -9.068 to 7.602 | No | ns | >0.9999 |
| FEW vs. NUK | -1.696 | -9.084 to 5.692 | No | ns | >0.9999 |
| FEW vs. GIG | -2.133 | -10.47 to 6.202 | No | ns | >0.9999 |
| FEW vs. BEM | -2.333 | -10.67 to 6.002 | No | ns | >0.9999 |
| FEW vs. PEF | -2.667 | -11.58 to 6.244 | No | ns | >0.9999 |
| FEW vs. LOT | -3.133 | -11.10 to 4.837 | No | ns | >0.9999 |
| FEW vs. BOON | -6.833 | -14.55 to 0.8835 | No | ns | 0.1814 |
| SAT vs. BOM | -0.3000 | -7.202 to 6.602 | No | ns | >0.9999 |
| SAT vs. TOP | -0.7000 | -8.021 to 6.621 | No | ns | >0.9999 |
| SAT vs. NUK | -1.663 | -7.884 to 4.559 | No | ns | >0.9999 |
| SAT vs. GIG | -2.100 | -9.421 to 5.221 | No | ns | >0.9999 |
| SAT vs. BEM | -2.300 | -9.621 to 5.021 | No | ns | >0.9999 |
| SAT vs. PEF | -2.633 | -10.60 to 5.337 | No | ns | >0.9999 |
| SAT vs. LOT | -3.100 | -10.00 to 3.802 | No | ns | >0.9999 |
| SAT vs. BOON | -6.800 | -13.41 to -0.1917 | Yes | * | 0.0357 |
| BOM vs. TOP | -0.4000 | -7.721 to 6.921 | No | ns | >0.9999 |
| BOM vs. NUK | -1.363 | -7.584 to 4.859 | No | ns | >0.9999 |
| BOM vs. GIG | -1.800 | -9.121 to 5.521 | No | ns | >0.9999 |
| BOM vs. BEM | -2.000 | -9.321 to 5.321 | No | ns | >0.9999 |
| BOM vs. PEF | -2.333 | -10.30 to 5.637 | No | ns | >0.9999 |
| BOM vs. LOT | -2.800 | -9.702 to 4.102 | No | ns | >0.9999 |
| BOM vs. BOON | -6.500 | -13.11 to 0.1083 | No | ns | 0.0604 |
| TOP vs. NUK | -0.9625 | -7.645 to 5.720 | No | ns | >0.9999 |
| TOP vs. GIG | -1.400 | -9.117 to 6.317 | No | ns | >0.9999 |
| TOP vs. BEM | -1.600 | -9.317 to 6.117 | No | ns | >0.9999 |
| TOP vs. PEF | -1.933 | -10.27 to 6.402 | No | ns | >0.9999 |
| TOP vs. LOT | -2.400 | -9.721 to 4.921 | No | ns | >0.9999 |
| TOP vs. BOON | -6.100 | -13.14 to 0.9445 | No | ns | 0.2243 |
| NUK vs. GIG | -0.4375 | -7.120 to 6.245 | No | ns | >0.9999 |
| NUK vs. BEM | -0.6375 | -7.320 to 6.045 | No | ns | >0.9999 |
| NUK vs. PEF | -0.9708 | -8.359 to 6.417 | No | ns | >0.9999 |
| NUK vs. LOT | -1.438 | -7.659 to 4.784 | No | ns | >0.9999 |
| NUK vs. BOON | -5.138 | -11.03 to 0.7563 | No | ns | 0.2108 |
| GIG vs. BEM | -0.2000 | -7.917 to 7.517 | No | ns | >0.9999 |
| GIG vs. PEF | -0.5333 | -8.868 to 7.802 | No | ns | >0.9999 |
| GIG vs. LOT | -1.000 | -8.321 to 6.321 | No | ns | >0.9999 |
| GIG vs. BOON | -4.700 | -11.74 to 2.344 | No | ns | >0.9999 |
| BEM vs. PEF | -0.3333 | -8.668 to 8.002 | No | ns | >0.9999 |
| BEM vs. LOT | -0.8000 | -8.121 to 6.521 | No | ns | >0.9999 |
| BEM vs. BOON | -4.500 | -11.54 to 2.544 | No | ns | >0.9999 |
| PEF vs. LOT | -0.4667 | -8.437 to 7.503 | No | ns | >0.9999 |
| PEF vs. BOON | -4.167 | -11.88 to 3.550 | No | ns | >0.9999 |
| LOT vs. BOON | -3.700 | -10.31 to 2.908 | No | ns | >0.9999 |

**Figure 1B**

| Number of families | 1 |  |  |  |  |
| --- | --- | --- | --- | --- | --- |
| Number of comparisons per family | 105 |  |  |  |  |
| Alpha | 0.05 |  |  |  |  |
|  |  |  |  |  |  |
| Bonferroni's multiple comparisons test | Mean Diff. | 95.00% CI of diff. | Significant? | Summary | Adjusted P Value |
| BOON vs. FEW | -1.182 | -2.798 to 0.4345 | No | ns | 0.9033 |
| BOON vs. DAVIS | -1.251 | -2.726 to 0.2248 | No | ns | 0.2731 |
| BOON vs. PEF | -1.565 | -3.181 to 0.05121 | No | ns | 0.0720 |
| BOON vs. LOX | -1.855 | -3.471 to -0.2388 | Yes | ** | 0.0086 |
| BOON vs. PIPING | -2.065 | -3.540 to -0.5893 | Yes | *** | 0.0004 |
| BOON vs. LAM | -2.133 | -3.608 to -0.6577 | Yes | *** | 0.0002 |
| BOON vs. BOM | -2.607 | -3.991 to -1.223 | Yes | **** | <0.0001 |
| BOON vs. GIG | -2.628 | -4.103 to -1.153 | Yes | **** | <0.0001 |
| BOON vs. SAT | -3.185 | -4.569 to -1.801 | Yes | **** | <0.0001 |
| BOON vs. LAT | -3.346 | -5.212 to -1.479 | Yes | **** | <0.0001 |
| BOON vs. BEM | -3.555 | -5.030 to -2.079 | Yes | **** | <0.0001 |
| BOON vs. LOT | -3.699 | -5.083 to -2.315 | Yes | **** | <0.0001 |
| BOON vs. TOP | -3.801 | -5.276 to -2.325 | Yes | **** | <0.0001 |
| BOON vs. NUK | -4.260 | -5.494 to -3.025 | Yes | **** | <0.0001 |
| FEW vs. DAVIS | -0.06889 | -1.815 to 1.677 | No | ns | >0.9999 |
| FEW vs. PEF | -0.3833 | -2.250 to 1.483 | No | ns | >0.9999 |
| FEW vs. LOX | -0.6733 | -2.540 to 1.193 | No | ns | >0.9999 |
| FEW vs. PIPING | -0.8831 | -2.629 to 0.8626 | No | ns | >0.9999 |
| FEW vs. LAM | -0.9514 | -2.697 to 0.7943 | No | ns | >0.9999 |
| FEW vs. BOM | -1.426 | -3.095 to 0.2437 | No | ns | 0.2550 |
| FEW vs. GIG | -1.446 | -3.192 to 0.2993 | No | ns | 0.3343 |
| FEW vs. SAT | -2.003 | -3.672 to -0.3337 | Yes | ** | 0.0045 |
| FEW vs. LAT | -2.164 | -4.250 to -0.07738 | Yes | * | 0.0324 |
| FEW vs. BEM | -2.373 | -4.119 to -0.6274 | Yes | *** | 0.0006 |
| FEW vs. LOT | -2.517 | -4.186 to -0.8477 | Yes | **** | <0.0001 |
| FEW vs. TOP | -2.619 | -4.365 to -0.8732 | Yes | **** | <0.0001 |
| FEW vs. NUK | -3.078 | -4.625 to -1.531 | Yes | **** | <0.0001 |
| DAVIS vs. PEF | -0.3144 | -2.060 to 1.431 | No | ns | >0.9999 |
| DAVIS vs. LOX | -0.6044 | -2.350 to 1.141 | No | ns | >0.9999 |
| DAVIS vs. PIPING | -0.8142 | -2.430 to 0.8020 | No | ns | >0.9999 |
| DAVIS vs. LAM | -0.8825 | -2.499 to 0.7337 | No | ns | >0.9999 |
| DAVIS vs. BOM | -1.357 | -2.890 to 0.1766 | No | ns | 0.1827 |
| DAVIS vs. GIG | -1.377 | -2.994 to 0.2387 | No | ns | 0.2597 |
| DAVIS vs. SAT | -1.934 | -3.467 to -0.4007 | Yes | ** | 0.0021 |
| DAVIS vs. LAT | -2.095 | -4.074 to -0.1156 | Yes | * | 0.0252 |
| DAVIS vs. BEM | -2.304 | -3.920 to -0.6880 | Yes | *** | 0.0003 |
| DAVIS vs. LOT | -2.448 | -3.981 to -0.9147 | Yes | **** | <0.0001 |
| DAVIS vs. TOP | -2.550 | -4.166 to -0.9338 | Yes | **** | <0.0001 |
| DAVIS vs. NUK | -3.009 | -4.409 to -1.609 | Yes | **** | <0.0001 |
| PEF vs. LOX | -0.2900 | -2.156 to 1.576 | No | ns | >0.9999 |
| PEF vs. PIPING | -0.4997 | -2.245 to 1.246 | No | ns | >0.9999 |
| PEF vs. LAM | -0.5681 | -2.314 to 1.178 | No | ns | >0.9999 |
| PEF vs. BOM | -1.042 | -2.711 to 0.6270 | No | ns | >0.9999 |
| PEF vs. GIG | -1.063 | -2.809 to 0.6826 | No | ns | >0.9999 |
| PEF vs. SAT | -1.620 | -3.289 to 0.04965 | No | ns | 0.0704 |
| PEF vs. LAT | -1.781 | -3.867 to 0.3060 | No | ns | 0.2568 |
| PEF vs. BEM | -1.990 | -3.735 to -0.2440 | Yes | ** | 0.0095 |
| PEF vs. LOT | -2.134 | -3.803 to -0.4643 | Yes | ** | 0.0017 |
| PEF vs. TOP | -2.236 | -3.981 to -0.4899 | Yes | ** | 0.0016 |
| PEF vs. NUK | -2.695 | -4.242 to -1.147 | Yes | **** | <0.0001 |
| LOX vs. PIPING | -0.2097 | -1.955 to 1.536 | No | ns | >0.9999 |
| LOX vs. LAM | -0.2781 | -2.024 to 1.468 | No | ns | >0.9999 |
| LOX vs. BOM | -0.7522 | -2.421 to 0.9170 | No | ns | >0.9999 |
| LOX vs. GIG | -0.7731 | -2.519 to 0.9726 | No | ns | >0.9999 |
| LOX vs. SAT | -1.330 | -2.999 to 0.3397 | No | ns | 0.4670 |
| LOX vs. LAT | -1.491 | -3.577 to 0.5960 | No | ns | >0.9999 |
| LOX vs. BEM | -1.700 | -3.445 to 0.04598 | No | ns | 0.0677 |
| LOX vs. LOT | -1.844 | -3.513 to -0.1743 | Yes | * | 0.0145 |
| LOX vs. TOP | -1.946 | -3.691 to -0.1999 | Yes | * | 0.0129 |
| LOX vs. NUK | -2.405 | -3.952 to -0.8573 | Yes | **** | <0.0001 |
| PIPING vs. LAM | -0.06833 | -1.685 to 1.548 | No | ns | >0.9999 |
| PIPING vs. BOM | -0.5425 | -2.076 to 0.9908 | No | ns | >0.9999 |
| PIPING vs. GIG | -0.5633 | -2.180 to 1.053 | No | ns | >0.9999 |
| PIPING vs. SAT | -1.120 | -2.653 to 0.4134 | No | ns | 0.9103 |
| PIPING vs. LAT | -1.281 | -3.260 to 0.6986 | No | ns | >0.9999 |
| PIPING vs. BEM | -1.490 | -3.106 to 0.1262 | No | ns | 0.1214 |
| PIPING vs. LOT | -1.634 | -3.167 to -0.1006 | Yes | * | 0.0232 |
| PIPING vs. TOP | -1.736 | -3.352 to -0.1196 | Yes | * | 0.0209 |
| PIPING vs. NUK | -2.195 | -3.595 to -0.7953 | Yes | **** | <0.0001 |
| LAM vs. BOM | -0.4742 | -2.007 to 1.059 | No | ns | >0.9999 |
| LAM vs. GIG | -0.4950 | -2.111 to 1.121 | No | ns | >0.9999 |
| LAM vs. SAT | -1.051 | -2.585 to 0.4818 | No | ns | >0.9999 |
| LAM vs. LAT | -1.212 | -3.192 to 0.7669 | No | ns | >0.9999 |
| LAM vs. BEM | -1.422 | -3.038 to 0.1945 | No | ns | 0.1934 |
| LAM vs. LOT | -1.565 | -3.099 to -0.03223 | Yes | * | 0.0392 |
| LAM vs. TOP | -1.667 | -3.284 to -0.05129 | Yes | * | 0.0345 |
| LAM vs. NUK | -2.127 | -3.526 to -0.7270 | Yes | **** | <0.0001 |
| BOM vs. GIG | -0.02083 | -1.554 to 1.512 | No | ns | >0.9999 |
| BOM vs. SAT | -0.5773 | -2.023 to 0.8682 | No | ns | >0.9999 |
| BOM vs. LAT | -0.7383 | -2.651 to 1.174 | No | ns | >0.9999 |
| BOM vs. BEM | -0.9475 | -2.481 to 0.5858 | No | ns | >0.9999 |
| BOM vs. LOT | -1.091 | -2.537 to 0.3542 | No | ns | 0.7130 |
| BOM vs. TOP | -1.193 | -2.727 to 0.3399 | No | ns | 0.5631 |
| BOM vs. NUK | -1.652 | -2.956 to -0.3495 | Yes | ** | 0.0019 |
| GIG vs. SAT | -0.5565 | -2.090 to 0.9768 | No | ns | >0.9999 |
| GIG vs. LAT | -0.7175 | -2.697 to 1.262 | No | ns | >0.9999 |
| GIG vs. BEM | -0.9267 | -2.543 to 0.6895 | No | ns | >0.9999 |
| GIG vs. LOT | -1.071 | -2.604 to 0.4628 | No | ns | >0.9999 |
| GIG vs. TOP | -1.173 | -2.789 to 0.4437 | No | ns | 0.9551 |
| GIG vs. NUK | -1.632 | -3.031 to -0.2320 | Yes | ** | 0.0069 |
| SAT vs. LAT | -0.1610 | -2.073 to 1.751 | No | ns | >0.9999 |
| SAT vs. BEM | -0.3702 | -1.903 to 1.163 | No | ns | >0.9999 |
| SAT vs. LOT | -0.5140 | -1.960 to 0.9316 | No | ns | >0.9999 |
| SAT vs. TOP | -0.6160 | -2.149 to 0.9173 | No | ns | >0.9999 |
| SAT vs. NUK | -1.075 | -2.378 to 0.2279 | No | ns | 0.3466 |
| LAT vs. BEM | -0.2092 | -2.189 to 1.770 | No | ns | >0.9999 |
| LAT vs. LOT | -0.3530 | -2.265 to 1.559 | No | ns | >0.9999 |
| LAT vs. TOP | -0.4550 | -2.434 to 1.524 | No | ns | >0.9999 |
| LAT vs. NUK | -0.9142 | -2.721 to 0.8928 | No | ns | >0.9999 |
| BEM vs. LOT | -0.1438 | -1.677 to 1.389 | No | ns | >0.9999 |
| BEM vs. TOP | -0.2458 | -1.862 to 1.370 | No | ns | >0.9999 |
| BEM vs. NUK | -0.7050 | -2.105 to 0.6947 | No | ns | >0.9999 |
| LOT vs. TOP | -0.1020 | -1.635 to 1.431 | No | ns | >0.9999 |
| LOT vs. NUK | -0.5612 | -1.864 to 0.7419 | No | ns | >0.9999 |
| TOP vs. NUK | -0.4592 | -1.859 to 0.9405 | No | ns | >0.9999 |

**Figure 1C**

| Number of families | 1 |  |  |  |  |
| --- | --- | --- | --- | --- | --- |
| Number of comparisons per family | 105 |  |  |  |  |
| Alpha | 0.05 |  |  |  |  |
|  |  |  |  |  |  |
| Bonferroni's multiple comparisons test | Mean Diff. | 95.00% CI of diff. | Significant? | Summary | Adjusted P Value |
| PEF vs. LOT | -59.71 | -1909 to 1790 | No | ns | >0.9999 |
| PEF vs. BOM | -93.68 | -2028 to 1841 | No | ns | >0.9999 |
| PEF vs. TOP | -139.2 | -2074 to 1795 | No | ns | >0.9999 |
| PEF vs. LOX | -145.3 | -2213 to 1923 | No | ns | >0.9999 |
| PEF vs. LAM | -303.6 | -2238 to 1631 | No | ns | >0.9999 |
| PEF vs. BOON | -392.9 | -2184 to 1398 | No | ns | >0.9999 |
| PEF vs. PIPING | -516.0 | -2450 to 1418 | No | ns | >0.9999 |
| PEF vs. LAT | -855.0 | -3167 to 1457 | No | ns | >0.9999 |
| PEF vs. DAVIS | -990.8 | -2925 to 943.5 | No | ns | >0.9999 |
| PEF vs. GIG | -1383 | -3318 to 551.0 | No | ns | >0.9999 |
| PEF vs. FEW | -1453 | -3521 to 614.7 | No | ns | >0.9999 |
| PEF vs. SAT | -1612 | -3461 to 238.0 | No | ns | 0.2110 |
| PEF vs. BEM | -1717 | -3651 to 217.3 | No | ns | 0.1767 |
| PEF vs. NUK | -2600 | -4314 to -885.2 | Yes | **** | <0.0001 |
| LOT vs. BOM | -33.97 | -1733 to 1665 | No | ns | >0.9999 |
| LOT vs. TOP | -79.50 | -1778 to 1619 | No | ns | >0.9999 |
| LOT vs. LOX | -85.62 | -1935 to 1764 | No | ns | >0.9999 |
| LOT vs. LAM | -243.9 | -1943 to 1455 | No | ns | >0.9999 |
| LOT vs. BOON | -333.2 | -1867 to 1200 | No | ns | >0.9999 |
| LOT vs. PIPING | -456.3 | -2155 to 1243 | No | ns | >0.9999 |
| LOT vs. LAT | -795.3 | -2914 to 1324 | No | ns | >0.9999 |
| LOT vs. DAVIS | -931.1 | -2630 to 767.9 | No | ns | >0.9999 |
| LOT vs. GIG | -1324 | -3022 to 375.4 | No | ns | 0.5564 |
| LOT vs. FEW | -1393 | -3243 to 456.1 | No | ns | 0.7209 |
| LOT vs. SAT | -1552 | -3154 to 49.94 | No | ns | 0.0715 |
| LOT vs. BEM | -1657 | -3356 to 41.61 | No | ns | 0.0662 |
| LOT vs. NUK | -2540 | -3984 to -1096 | Yes | **** | <0.0001 |
| BOM vs. TOP | -45.53 | -1836 to 1745 | No | ns | >0.9999 |
| BOM vs. LOX | -51.65 | -1986 to 1883 | No | ns | >0.9999 |
| BOM vs. LAM | -209.9 | -2001 to 1581 | No | ns | >0.9999 |
| BOM vs. BOON | -299.2 | -1934 to 1336 | No | ns | >0.9999 |
| BOM vs. PIPING | -422.3 | -2213 to 1368 | No | ns | >0.9999 |
| BOM vs. LAT | -761.4 | -2955 to 1432 | No | ns | >0.9999 |
| BOM vs. DAVIS | -897.1 | -2688 to 893.7 | No | ns | >0.9999 |
| BOM vs. GIG | -1290 | -3080 to 501.2 | No | ns | >0.9999 |
| BOM vs. FEW | -1360 | -3294 to 574.8 | No | ns | >0.9999 |
| BOM vs. SAT | -1518 | -3217 to 181.1 | No | ns | 0.1659 |
| BOM vs. BEM | -1623 | -3414 to 167.5 | No | ns | 0.1437 |
| BOM vs. NUK | -2506 | -4057 to -955.2 | Yes | **** | <0.0001 |
| TOP vs. LOX | -6.125 | -1940 to 1928 | No | ns | >0.9999 |
| TOP vs. LAM | -164.4 | -1955 to 1626 | No | ns | >0.9999 |
| TOP vs. BOON | -253.7 | -1888 to 1381 | No | ns | >0.9999 |
| TOP vs. PIPING | -376.8 | -2168 to 1414 | No | ns | >0.9999 |
| TOP vs. LAT | -715.8 | -2909 to 1477 | No | ns | >0.9999 |
| TOP vs. DAVIS | -851.6 | -2642 to 939.2 | No | ns | >0.9999 |
| TOP vs. GIG | -1244 | -3035 to 546.7 | No | ns | >0.9999 |
| TOP vs. FEW | -1314 | -3248 to 620.3 | No | ns | >0.9999 |
| TOP vs. SAT | -1472 | -3171 to 226.6 | No | ns | 0.2220 |
| TOP vs. BEM | -1578 | -3369 to 213.0 | No | ns | 0.1899 |
| TOP vs. NUK | -2461 | -4011 to -909.7 | Yes | **** | <0.0001 |
| LOX vs. LAM | -158.3 | -2093 to 1776 | No | ns | >0.9999 |
| LOX vs. BOON | -247.5 | -2038 to 1543 | No | ns | >0.9999 |
| LOX vs. PIPING | -370.7 | -2305 to 1564 | No | ns | >0.9999 |
| LOX vs. LAT | -709.7 | -3022 to 1602 | No | ns | >0.9999 |
| LOX vs. DAVIS | -845.5 | -2780 to 1089 | No | ns | >0.9999 |
| LOX vs. GIG | -1238 | -3172 to 696.4 | No | ns | >0.9999 |
| LOX vs. FEW | -1308 | -3376 to 760.0 | No | ns | >0.9999 |
| LOX vs. SAT | -1466 | -3316 to 383.4 | No | ns | 0.4837 |
| LOX vs. BEM | -1572 | -3506 to 362.6 | No | ns | 0.3940 |
| LOX vs. NUK | -2454 | -4169 to -739.9 | Yes | *** | 0.0002 |
| LAM vs. BOON | -89.28 | -1724 to 1546 | No | ns | >0.9999 |
| LAM vs. PIPING | -212.4 | -2003 to 1578 | No | ns | >0.9999 |
| LAM vs. LAT | -551.5 | -2745 to 1642 | No | ns | >0.9999 |
| LAM vs. DAVIS | -687.2 | -2478 to 1104 | No | ns | >0.9999 |
| LAM vs. GIG | -1080 | -2871 to 711.1 | No | ns | >0.9999 |
| LAM vs. FEW | -1150 | -3084 to 784.7 | No | ns | >0.9999 |
| LAM vs. SAT | -1308 | -3007 to 391.0 | No | ns | 0.6109 |
| LAM vs. BEM | -1413 | -3204 to 377.4 | No | ns | 0.5012 |
| LAM vs. NUK | -2296 | -3847 to -745.3 | Yes | *** | 0.0001 |
| BOON vs. PIPING | -123.1 | -1758 to 1512 | No | ns | >0.9999 |
| BOON vs. LAT | -462.2 | -2530 to 1606 | No | ns | >0.9999 |
| BOON vs. DAVIS | -597.9 | -2233 to 1037 | No | ns | >0.9999 |
| BOON vs. GIG | -990.4 | -2625 to 644.4 | No | ns | >0.9999 |
| BOON vs. FEW | -1060 | -2851 to 730.5 | No | ns | >0.9999 |
| BOON vs. SAT | -1219 | -2752 to 314.9 | No | ns | 0.4742 |
| BOON vs. BEM | -1324 | -2959 to 310.6 | No | ns | 0.4046 |
| BOON vs. NUK | -2207 | -3575 to -839.2 | Yes | **** | <0.0001 |
| PIPING vs. LAT | -339.0 | -2532 to 1854 | No | ns | >0.9999 |
| PIPING vs. DAVIS | -474.8 | -2266 to 1316 | No | ns | >0.9999 |
| PIPING vs. GIG | -867.3 | -2658 to 923.5 | No | ns | >0.9999 |
| PIPING vs. FEW | -937.2 | -2872 to 997.1 | No | ns | >0.9999 |
| PIPING vs. SAT | -1096 | -2794 to 603.4 | No | ns | >0.9999 |
| PIPING vs. BEM | -1201 | -2992 to 589.8 | No | ns | >0.9999 |
| PIPING vs. NUK | -2084 | -3635 to -532.9 | Yes | *** | 0.0007 |
| LAT vs. DAVIS | -135.8 | -2329 to 2058 | No | ns | >0.9999 |
| LAT vs. GIG | -528.3 | -2722 to 1665 | No | ns | >0.9999 |
| LAT vs. FEW | -598.2 | -2910 to 1714 | No | ns | >0.9999 |
| LAT vs. SAT | -756.5 | -2875 to 1362 | No | ns | >0.9999 |
| LAT vs. BEM | -862.0 | -3055 to 1331 | No | ns | >0.9999 |
| LAT vs. NUK | -1745 | -3747 to 257.5 | No | ns | 0.2108 |
| DAVIS vs. GIG | -392.5 | -2183 to 1398 | No | ns | >0.9999 |
| DAVIS vs. FEW | -462.4 | -2397 to 1472 | No | ns | >0.9999 |
| DAVIS vs. SAT | -620.8 | -2320 to 1078 | No | ns | >0.9999 |
| DAVIS vs. BEM | -726.3 | -2517 to 1065 | No | ns | >0.9999 |
| DAVIS vs. NUK | -1609 | -3160 to -58.10 | Yes | * | 0.0323 |
| GIG vs. FEW | -69.92 | -2004 to 1864 | No | ns | >0.9999 |
| GIG vs. SAT | -228.3 | -1927 to 1471 | No | ns | >0.9999 |
| GIG vs. BEM | -333.8 | -2125 to 1457 | No | ns | >0.9999 |
| GIG vs. NUK | -1217 | -2767 to 334.4 | No | ns | 0.5270 |
| FEW vs. SAT | -158.3 | -2008 to 1691 | No | ns | >0.9999 |
| FEW vs. BEM | -263.8 | -2198 to 1670 | No | ns | >0.9999 |
| FEW vs. NUK | -1147 | -2861 to 568.0 | No | ns | >0.9999 |
| SAT vs. BEM | -105.5 | -1804 to 1593 | No | ns | >0.9999 |
| SAT vs. NUK | -988.3 | -2432 to 455.6 | No | ns | >0.9999 |
| BEM vs. NUK | -882.8 | -2434 to 668.1 | No | ns | >0.9999 |

**Figure 1D**

| Number of families | 1 |  |  |  |  |
| --- | --- | --- | --- | --- | --- |
| Number of comparisons per family | 91 |  |  |  |  |
| Alpha | 0.05 |  |  |  |  |
|  |  |  |  |  |  |
| Bonferroni's multiple comparisons test | Mean Diff. | 95.00% CI of diff. | Significant? | Summary | Adjusted P Value |
| BEM vs. DAVIS | 41.20 | -63.71 to 146.1 | No | ns | >0.9999 |
| BEM vs. LOT | -18.32 | -130.5 to 93.83 | No | ns | >0.9999 |
| BEM vs. LAT | 30.80 | -94.59 to 156.2 | No | ns | >0.9999 |
| BEM vs. SAT | 28.41 | -71.90 to 128.7 | No | ns | >0.9999 |
| BEM vs. NUK | -9.318 | -104.1 to 85.47 | No | ns | >0.9999 |
| BEM vs. LAM | 8.249 | -96.66 to 113.2 | No | ns | >0.9999 |
| BEM vs. GIG | 6.593 | -98.32 to 111.5 | No | ns | >0.9999 |
| BEM vs. PIPING | 6.030 | -98.88 to 110.9 | No | ns | >0.9999 |
| BEM vs. FEW | -1.965 | -114.1 to 110.2 | No | ns | >0.9999 |
| BEM vs. TOP | -3.255 | -108.2 to 101.7 | No | ns | >0.9999 |
| BEM vs. BOM | -11.38 | -123.5 to 100.8 | No | ns | >0.9999 |
| BEM vs. PEF | -11.94 | -124.1 to 100.2 | No | ns | >0.9999 |
| BEM vs. BOON | -22.39 | -119.5 to 74.74 | No | ns | >0.9999 |
| DAVIS vs. LOT | -59.52 | -164.4 to 45.39 | No | ns | >0.9999 |
| DAVIS vs. LAT | -10.40 | -129.4 to 108.6 | No | ns | >0.9999 |
| DAVIS vs. SAT | -12.79 | -104.9 to 79.36 | No | ns | >0.9999 |
| DAVIS vs. NUK | -50.51 | -136.6 to 35.58 | No | ns | >0.9999 |
| DAVIS vs. LAM | -32.95 | -130.1 to 64.18 | No | ns | >0.9999 |
| DAVIS vs. GIG | -34.60 | -131.7 to 62.53 | No | ns | >0.9999 |
| DAVIS vs. PIPING | -35.17 | -132.3 to 61.96 | No | ns | >0.9999 |
| DAVIS vs. FEW | -43.16 | -148.1 to 61.75 | No | ns | >0.9999 |
| DAVIS vs. TOP | -44.45 | -141.6 to 52.68 | No | ns | >0.9999 |
| DAVIS vs. BOM | -52.58 | -157.5 to 52.33 | No | ns | >0.9999 |
| DAVIS vs. PEF | -53.13 | -158.0 to 51.78 | No | ns | >0.9999 |
| DAVIS vs. BOON | -63.59 | -152.3 to 25.08 | No | ns | 0.9404 |
| LOT vs. LAT | 49.12 | -76.27 to 174.5 | No | ns | >0.9999 |
| LOT vs. SAT | 46.73 | -53.58 to 147.0 | No | ns | >0.9999 |
| LOT vs. NUK | 9.006 | -85.78 to 103.8 | No | ns | >0.9999 |
| LOT vs. LAM | 26.57 | -78.34 to 131.5 | No | ns | >0.9999 |
| LOT vs. GIG | 24.92 | -79.99 to 129.8 | No | ns | >0.9999 |
| LOT vs. PIPING | 24.35 | -80.56 to 129.3 | No | ns | >0.9999 |
| LOT vs. FEW | 16.36 | -95.80 to 128.5 | No | ns | >0.9999 |
| LOT vs. TOP | 15.07 | -89.84 to 120.0 | No | ns | >0.9999 |
| LOT vs. BOM | 6.943 | -105.2 to 119.1 | No | ns | >0.9999 |
| LOT vs. PEF | 6.387 | -105.8 to 118.5 | No | ns | >0.9999 |
| LOT vs. BOON | -4.067 | -101.2 to 93.06 | No | ns | >0.9999 |
| LAT vs. SAT | -2.389 | -117.3 to 112.5 | No | ns | >0.9999 |
| LAT vs. NUK | -40.12 | -150.3 to 70.02 | No | ns | >0.9999 |
| LAT vs. LAM | -22.55 | -141.5 to 96.41 | No | ns | >0.9999 |
| LAT vs. GIG | -24.21 | -143.2 to 94.75 | No | ns | >0.9999 |
| LAT vs. PIPING | -24.77 | -143.7 to 94.19 | No | ns | >0.9999 |
| LAT vs. FEW | -32.77 | -158.2 to 92.63 | No | ns | >0.9999 |
| LAT vs. TOP | -34.06 | -153.0 to 84.90 | No | ns | >0.9999 |
| LAT vs. BOM | -42.18 | -167.6 to 83.21 | No | ns | >0.9999 |
| LAT vs. PEF | -42.74 | -168.1 to 82.66 | No | ns | >0.9999 |
| LAT vs. BOON | -53.19 | -165.3 to 58.96 | No | ns | >0.9999 |
| SAT vs. NUK | -37.73 | -118.2 to 42.70 | No | ns | >0.9999 |
| SAT vs. LAM | -20.16 | -112.3 to 71.98 | No | ns | >0.9999 |
| SAT vs. GIG | -21.82 | -114.0 to 70.33 | No | ns | >0.9999 |
| SAT vs. PIPING | -22.38 | -114.5 to 69.76 | No | ns | >0.9999 |
| SAT vs. FEW | -30.38 | -130.7 to 69.94 | No | ns | >0.9999 |
| SAT vs. TOP | -31.67 | -123.8 to 60.48 | No | ns | >0.9999 |
| SAT vs. BOM | -39.79 | -140.1 to 60.52 | No | ns | >0.9999 |
| SAT vs. PEF | -40.35 | -140.7 to 59.97 | No | ns | >0.9999 |
| SAT vs. BOON | -50.80 | -134.0 to 32.38 | No | ns | >0.9999 |
| NUK vs. LAM | 17.57 | -68.53 to 103.7 | No | ns | >0.9999 |
| NUK vs. GIG | 15.91 | -70.18 to 102.0 | No | ns | >0.9999 |
| NUK vs. PIPING | 15.35 | -70.75 to 101.4 | No | ns | >0.9999 |
| NUK vs. FEW | 7.352 | -87.44 to 102.1 | No | ns | >0.9999 |
| NUK vs. TOP | 6.062 | -80.03 to 92.16 | No | ns | >0.9999 |
| NUK vs. BOM | -2.063 | -96.85 to 92.73 | No | ns | >0.9999 |
| NUK vs. PEF | -2.619 | -97.41 to 92.17 | No | ns | >0.9999 |
| NUK vs. BOON | -13.07 | -89.49 to 63.35 | No | ns | >0.9999 |
| LAM vs. GIG | -1.655 | -98.78 to 95.47 | No | ns | >0.9999 |
| LAM vs. PIPING | -2.218 | -99.35 to 94.91 | No | ns | >0.9999 |
| LAM vs. FEW | -10.21 | -115.1 to 94.70 | No | ns | >0.9999 |
| LAM vs. TOP | -11.50 | -108.6 to 85.63 | No | ns | >0.9999 |
| LAM vs. BOM | -19.63 | -124.5 to 85.28 | No | ns | >0.9999 |
| LAM vs. PEF | -20.18 | -125.1 to 84.73 | No | ns | >0.9999 |
| LAM vs. BOON | -30.64 | -119.3 to 58.03 | No | ns | >0.9999 |
| GIG vs. PIPING | -0.5629 | -97.69 to 96.57 | No | ns | >0.9999 |
| GIG vs. FEW | -8.558 | -113.5 to 96.35 | No | ns | >0.9999 |
| GIG vs. TOP | -9.848 | -107.0 to 87.28 | No | ns | >0.9999 |
| GIG vs. BOM | -17.97 | -122.9 to 86.94 | No | ns | >0.9999 |
| GIG vs. PEF | -18.53 | -123.4 to 86.38 | No | ns | >0.9999 |
| GIG vs. BOON | -28.98 | -117.6 to 59.68 | No | ns | >0.9999 |
| PIPING vs. FEW | -7.995 | -112.9 to 96.92 | No | ns | >0.9999 |
| PIPING vs. TOP | -9.285 | -106.4 to 87.84 | No | ns | >0.9999 |
| PIPING vs. BOM | -17.41 | -122.3 to 87.50 | No | ns | >0.9999 |
| PIPING vs. PEF | -17.97 | -122.9 to 86.94 | No | ns | >0.9999 |
| PIPING vs. BOON | -28.42 | -117.1 to 60.25 | No | ns | >0.9999 |
| FEW vs. TOP | -1.290 | -106.2 to 103.6 | No | ns | >0.9999 |
| FEW vs. BOM | -9.415 | -121.6 to 102.7 | No | ns | >0.9999 |
| FEW vs. PEF | -9.971 | -122.1 to 102.2 | No | ns | >0.9999 |
| FEW vs. BOON | -20.42 | -117.6 to 76.70 | No | ns | >0.9999 |
| TOP vs. BOM | -8.125 | -113.0 to 96.79 | No | ns | >0.9999 |
| TOP vs. PEF | -8.681 | -113.6 to 96.23 | No | ns | >0.9999 |
| TOP vs. BOON | -19.13 | -107.8 to 69.53 | No | ns | >0.9999 |
| BOM vs. PEF | -0.5558 | -112.7 to 111.6 | No | ns | >0.9999 |
| BOM vs. BOON | -11.01 | -108.1 to 86.12 | No | ns | >0.9999 |
| PEF vs. BOON | -10.45 | -107.6 to 86.68 | No | ns | >0.9999 |
